# Association between the Carbohydrate Quality Index (CQI) and Nutritional Adequacy in a Pediatric Cohort: The SENDO Project

**DOI:** 10.3390/children10101711

**Published:** 2023-10-20

**Authors:** Elise Fabios, Miguel Ángel Martínez-González, Lorena García-Blanco, Víctor de la O, Susana Santiago, Itziar Zazpe, Nerea Martín-Calvo

**Affiliations:** 1 Department of Preventive Medicine and Public Health, School of Medicine, University of Navarra, 31008 Pamplona, Spain; efabios@unav.es (E.F.); vdelao@unav.es (V.d.l.O.); izazpe@unav.es (I.Z.); nmartincalvo@unav.es (N.M.-C.); 2 IdiSNA, Instituto de Investigación Sanitaria de Navarra, 31008 Pamplona, Spain; ssantiago@unav.es; 3 Biomedical Research Centre Network on Obesity and Nutrition (CIBERobn), Physiopathology of Obesity and Nutrition, Institute of Health Carlos III, 28029 Madrid, Spain; 4 San Juan Primary Care Health Center, Servicio Navarro de Salud-Osasunbidea, 31011 Pamplona, Spain; lorena.garcia.blanco@navarra.es; 5 Precision Nutrition and Cardiometabolic Health Program, IMDEA Food, 28049 Madrid, Spain; 6 Department of Nutrition, Food Science and Physiology, School of Pharmacy, University of Navarra, 31008 Pamplona, Spain

**Keywords:** carbohydrate quality index, CQI, micronutrient inadequacy, SENDO cohort

## Abstract

Suboptimal micronutrient intake in children remains a public health concern around the world. This study examined the relationship between a previously defined dietary carbohydrate quality index (CQI) and the risk of micronutrient intake inadequacy in a pediatric cohort of Spanish preschoolers. Children aged 4–5 years old were recruited at their medical center or at school, and information on sociodemographic, dietary, and lifestyle variables were collected through a self-administered online questionnaire. Dietary information was obtained from a validated 147-item semi-quantitative food frequency questionnaire. We calculated the CQI and categorized participants into quartiles according to their scores. We assessed the intakes of 20 micronutrients and evaluated the probability of intake inadequacy by using the estimated average requirement cut-off point. Generalized estimating equations were used to adjust for potential confounders and account for the intra-cluster correlations between siblings. The adjusted proportions of children with an inadequate intake of ≥three micronutrients were 23%, 12%, 11%, and 9% in the first, second, third, and fourth quartiles of the CQI, respectively. Children in the highest quartile of the CQI had 0.22-fold lower odds (95% CI 0.10–0.48) of having ≥three inadequate micronutrient intakes than their peers in the lowest quartile. These findings reinforce the relevance of carbohydrate quality in children’s diets.

## 1. Introduction

Micronutrients, also known as vitamins and minerals, are an essential part of a healthy diet and contribute to optimal health. They are involved in numerous physiological processes, playing a key role in the production and proper functioning of enzymes, hormones and other molecules required for the body’s normal growth and development [1]. Even though micronutrients are needed in small quantities, an inadequate intake can seriously compromise health [2,3,4,5].

Micronutrients are especially important in the most vulnerable stages of life, such as childhood, due to the higher demands required by children’s rapid growth and development. Despite numerous advances and improvements in child health, malnutrition and concomitant micronutrient deficiencies continue to be a major concern around the world, especially in developing nations [2,6]. They also remain a public health challenge in more affluent countries. Several studies across Europe and America have shown that there is still a high prevalence of suboptimal micronutrient intake among children and adolescents [7,8,9,10].

On the other hand, food-based approaches are considered a long-term strategy for improving nutrition in order to meet nutritional requirements. In this sense, a growing number of voices are calling for dietary guidelines and policies to put emphasis on the quality of macronutrients in the diet rather than on the quantity [11,12,13]. Evidence has shown that consuming significant amounts of low-quality carbohydrates, such as refined grains or foods rich in added sugars, can quickly account for a person’s daily energetic needs, potentially displacing the consumption of other nutrient-dense foods. In this sense, studies on children and adolescents have concluded that the intake of numerous nutrients does in fact decrease significantly in diets high in added sugars and as the energy contribution of ultra-processed foods increases [14,15,16,17,18].

Other characteristics of carbohydrates have gained relevance in relation to health and can be useful in assessing carbohydrate quality. These include measures of whole grain consumption, dietary fiber, fiber from cereal, glycemic index (GI), and ratios such as carbohydrate-to-fiber and carbohydrate-to-cereal fiber. A growing body of evidence shows that carbohydrate quality is in fact a more important determinant of health than carbohydrate quantity alone [19,20,21,22,23], especially in relation to cardiometabolic risk factors.

However, one-dimension indicators may not fully grasp the complex and multiple effects of carbohydrate quality on health [24]. A more comprehensive approach, which combines different aspects of carbohydrate quality, might be more suitable for assessing this relationship. The carbohydrate quality index (CQI) is a new method, first proposed in 2014 by Zazpe et al. [22], that combines four aspects of carbohydrate quality: a low GI, high amount of dietary fiber, high ratio of whole grain to total grain (whole plus refined grain), and high ratio of solid to total carbohydrate (solid plus liquid). Each of these four components is weighted positively except for the GI. For each of these components the participants are categorized into quintiles and given a value ranging from 1 to 5 according to the quintile that they are in. The CQI is constructed by adding up all of these values, with the final score ranging from 4 to 20, with higher values corresponding to a better quality of carbohydrates (Table S1).

In previous observational studies in adults, diets with a higher CQI have been associated with rapid improvements in cardiovascular risk factors, including body weight, visceral fat and waist circumference [23,25], a lower risk of metabolic syndrome [26], gestational diabetes mellitus [27], breast cancer [28], colorectal cancer [29], and depression [30], among other health outcomes. Regarding micronutrient adequacy, three previous studies on adults reported a strong association of the CQI with nutritional adequacy [22,31,32].

To our knowledge, no study has been conducted on children to determine if the quality of dietary carbohydrates is associated with nutritional adequacy. Therefore, we investigated the association between carbohydrate quality, measured with the CQI, and overall nutritional adequacy, considering 20 micronutrients, in a pediatric population of Spanish children.

## 2. Materials and Methods

### 2.1. Study Population

The Seguimiento del Niño para un Desarrollo Óptimo (SENDO) project is an open, multidisciplinary, multiple-outcome, and prospective cohort study on Spanish children, designed to analyze the effect of diet and lifestyle on the health of children and adolescents. The ongoing recruitment began in 2015 and participants were invited to join by their pediatrician at their primary care center or by the SENDO team of researchers through schools. The inclusion criteria included being a child aged between 4 and 5 years old as well as being a Spanish resident. The only exclusion criterion was the impossibility of accessing a device connected to the Internet to fulfill the online questionnaires. Information was collected at baseline and updated every year through online self-administered questionnaires which were completed by the child’s parents.

**Figure 1:**
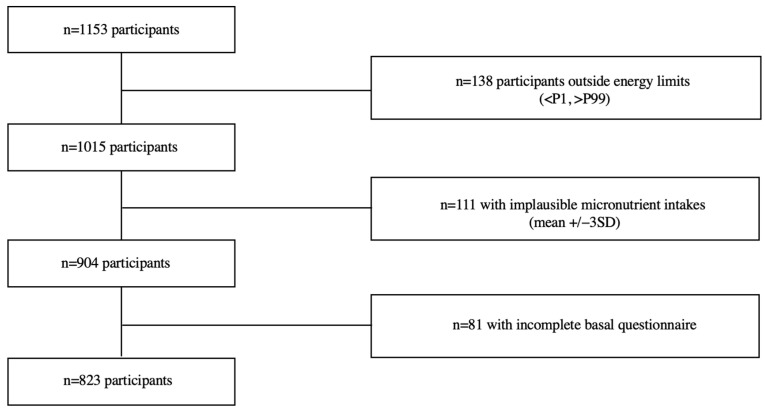
Flow chart of participants recruited in the SENDO project, 2014–2022.

In this cross-sectional study, we used the baseline information of participants recruited between January 2015 and June 2022. Of the 1157 participants recruited before June 2022, we excluded participants who reported a total energy intake outside predefined limits (<P1 or >P99) (n = 138) and those with micronutrients intakes ≥ 3 standard deviations (SDs) apart from the mean (n = 111). Of those remaining, 81 participants had an incomplete baseline questionnaire and were consequently excluded (see Figure 1). Thus, the final sample consisted of 823 participants.

The SENDO project follows the rules of the Declaration of Helsinki on the ethical principles for medical research on human beings. This study was approved by the Ethics Committee for Clinical Research of Navarra (Pyto 2016/122). Informed consent was obtained from all of the participants’ parents during recruitment.

### 2.2. Dietary Assessment

Dietary information was collected through a previously validated 147-item semi-quantitative food frequency questionnaire (FFQ) [33]. A portion size was specified for each food item. Parents reported how often their child had consumed each of the food items over the previous year by choosing one out of nine frequencies of consumption, ranging from “never or almost never” to “≥6 times per day”. The nutrient content of the food items was calculated by trained dieticians by multiplying the edible portion by the frequency of consumption and the nutrient composition of the specified portion size. Updated Spanish food composition tables and online databases were used for this purpose [34].

As previously described, the CQI of each participant was defined based on the following four criteria: the GI; the dietary fiber intake; the ratio of whole grains:total grains; and, finally, the ratio of solid carbohydrates:total carbohydrates (Table S1).

The dietary fiber intake was measured in grams per day. GI values were taken from the updated tables published by Atkinson et al. in 2021 [35], and a mean GI was calculated for each participant. This was carried out by adding the glycemic load contribution of each food consumed on average daily and dividing it by the total amount of carbohydrates consumed on average daily. For the third criteria, the intake of total grains was calculated by adding the intakes of whole grains (calculated based on the consumption of whole bread), refined grains, and their respective byproducts. Finally, solid carbohydrate content was calculated by comparing liquid carbohydrates to the total carbohydrate content in the diet. Sources of liquid carbohydrates included sugar-sweetened beverages and fruit juices.

For each of these 4 components, participants were categorized into quintiles and were given a value (ranging from 1 to 5) according to each quintile. All were positively weighted except for the GI. The CQI was constructed by adding up all of these values, with the final score ranging from 4 to 20, with higher values corresponding to a better quality of carbohydrates (Table S1). Lastly, the participants were divided into quartiles according to their CQI values.

We also assessed the consumption of food from 15 different food groups: vegetables, fruits, legumes, dairy, cereals, potatoes, meat, fish, nuts, bakery and sweets, beverages, fast food, eggs, olive oil, and other types of oils and fats (sunflower oil, butter, and mayonnaise).

Lastly, we calculated the KIDMED score of each participant to assess their adherence to the Mediterranean diet. This score has been described previously [36].

### 2.3. Assessment of Covariates

The baseline questionnaire collected information on family and personal medical histories, dietary habits, and sociodemographic as well as lifestyle variables.

The child’s and mother’s ages were calculated as the difference between the date on which the questionnaire was received and their respective birth date. Body mass index (BMI) was calculated as the ratio between reported weight (kg) and height squared (m^2^). Information on the standard procedures with which to collect these data was indicated in the questionnaire. Nutritional status was defined using sex- and age-specific cut-off points for the BMI based on the International Obesity Task Force standards of reference [37]. Age- and sex-specific z-scores of the BMI were calculated using the LMS method [37]. Anthropometric data was validated in a separate study, showing a high correlation and concordance between the data collected in the physical exam and that reported by the parents [38].

Physical activity was assessed with a questionnaire that included 14 activities and 10 answer options, from never to 11 or more hours/week. Of these, 4 activities were considered as moderate (≤5 METs/hour) and 10 as intense (>5 METs/hour). Participants indicated the average time dedicated to each activity during the previous year and we calculated the annual mean hours per day spent doing moderate–vigorous activities.

Screen time was calculated as the mean of hours per day spent watching television, using a computer, or playing video games. Time spent on weekdays and weekends was assessed separately.

Parental attitudes towards their child’s dietary habits were evaluated through 8 yes/no questions. Affirmative answers (i.e., healthy attitudes) were assigned 1 point and negative answer 0 points. Hence, the final score ranged from 0 to 8 points, with a higher score suggesting healthier attitudes. The participants’ parents were classified as having unhealthy (0–3 points), medium (4–6 points), or healthy attitudes (7–8 points) towards their child’s dietary habits.

Parental knowledge on nutritional recommendations for children was evaluated with questions on the recommended intake frequency of 18 different food groups and with 9 categories of response, ranging from “never” to “≥6 times per day”. Each question was assigned 1 point if the answer matched the dietary recommendations and 0 points if it did not. The final score was expressed as a percentage, with higher values meaning greater knowledge on nutritional recommendations for children. The participants’ parents were categorized as having high (>70%), medium (40–70%), or low (<40%) nutritional knowledge. The parental attitudes and parental knowledge scores have been used in previous studies of the SENDO project [39].

### 2.4. Outcome Assessment

We aimed to determine the micronutrient intake adequacy for the following 20 micronutrients with known public health relevance: vitamin A; vitamin C; vitamin D; vitamin E; vitamin B1; vitamin B2; vitamin B3; vitamin B6; folic acid; vitamin B12; Ca; I; Fe; P; Mg; Se; Zn; Cr; K; and Na. The prevalence of intake adequacy was calculated by comparing the intakes of these nutrients with the estimated average requirements (EARs) when these were available or adequate intake levels (AIs) if not, as proposed by the Institute of Medicine [40,41,42,43,44,45,46].

We also performed a sensitivity analysis by adding the participants’ intake of supplements over the previous year to the total intake of micronutrients.

### 2.5. Statistical Analysis

We described the participants’ characteristics by quartiles of the CQI. For descriptive purposes, we used percentages for categorical variables and means (SDs) for continuous variables. Linear trend tests across quartiles of the CQI were calculated by assigning the median of the CQI to each quartile and treating this variable as continuous in regression models. Linear trend Chi-squared tests were used for categorical variables.

In the main analyses we calculated (1) the difference and 95% confidence interval (CI) in the number of inadequate intakes of micronutrients across values of the CQI and (2) the odds ratio (OR) and 95% CI for the inadequate intake of ≥3 micronutrients associated with the CQI. Crude and multivariable-adjusted estimates were calculated. Multivariate analyses were progressively adjusted for (1) sex (female vs. male), age (continuous), nutritional status (low weight, normal weight, or overweight/obese), and energy intake (continuous); (2) maternal age (continuous), number of children (1, 2, 3–4, 5 or more), breastfeeding duration (none, <6 months, 6–12 months, and >12 months)), birth weight (<2500 g, 2500–3000 g, 3000–3500 g, 3500–4000 g, and >4000 g), parental knowledge about nutritional recommendations for children (low, medium, or high score), and parental attitudes towards the child’s dietary habits (low, medium, or high score); and (3) moderate–vigorous physical activity (continuous) and screen time (continuous).

In further analyses we calculated the marginal effect of the CQI on the risk of having ≥3 inadequate intakes of micronutrients; that is, the adjusted proportion of children with an inadequate intake of ≥3 micronutrients in each quartile of the CQI [47].

To assess the magnitude of potential residual confounding, we calculated the E-value as proposed by VanderWeele et al. [48].

We fitted generalized estimating equations to account for intra-cluster correlation between siblings. Analyses were carried out using Stata version 15.0 (STATA Corp., College Station, TX, USA). All *p*-values are two-tailed. Statistical significance was set at the conventional cut-off point of *p* < 0.05.

## 3. Results

The present study included 823 participants (49% of which were girls) aged 5.00 (SD: 0.84) years on average. The median CQI was 11 (interquartile range (IQR): 9–14). The main characteristics of the study participants and their parents by quartiles of the CQI are presented in Table 1.

In relation to family factors, we found that children with lower scores of the CQI were more likely to belong to more numerous families (*p* < 0.001) and to have parents with less knowledge of children’s nutritional requirements (*p* < 0.001) as well as less healthy attitudes towards their child’s dietary habits (*p* < 0.001). Regarding children’s characteristics, we observed that those with higher CQI scores were more physically active (*p* < 0.001) and were more likely to have been breastfed (*p* < 0.001). Regarding nutritional status, a marginally significant inverse association was observed between nutritional status and the CQI, with a smaller proportion of overweight and obese children in the quartile with higher CQI scores.

Regarding diet composition, as shown in Table 2, participants with higher scores of the CQI (fourth quartile) presented higher consumption of fruits, vegetables, legumes, and nuts (*p* for trend < 0.001), and a lower consumption of sugar-sweetened beverages (*p* for trend < 0.001).

As shown in Table 2, the percentages of total energy intake from carbohydrates across the different quartiles of the CQI were fairly constant, and ranged from 42.47 to 44.60% (*p* for trend < 0.001). The percentage of total energy represented by protein ranged from 16.84 to 17.20% (*p* for trend = 0.40). The percentage of total energy represented by fat ranged from 38.56 to 40.44% (*p* for trend < 0.001).

Table S2 shows the mean values of the different parameters of carbohydrate quality intake according to quartiles of the overall CQI. Participants with higher scores of the CQI had higher intakes of fiber, solid carbohydrates, and whole grains, a lower intake of liquid carbohydrates and refined grains, and a lower GI.

We calculated the energy-adjusted mean intake of each micronutrient by quartiles of the CQI. The results are displayed in Table 3. We found significant positive linear trends between the CQI and the intake of 15 out of the 20 micronutrients evaluated, including vitamin A, vitamin C, vitamin D, vitamin E, vitamin B1, vitamin B3, vitamin B6, folate, Fe, P, Mg, Se, Zn, Cr, and K. The nutrients that had a higher prevalence of inadequacy were K, vitamin E, folate, Ca, and vitamin D (Table S3). The prevalence of inadequate micronutrient intake according to quartiles of the CQI is summarized in Table S3.

**Figure 2:**
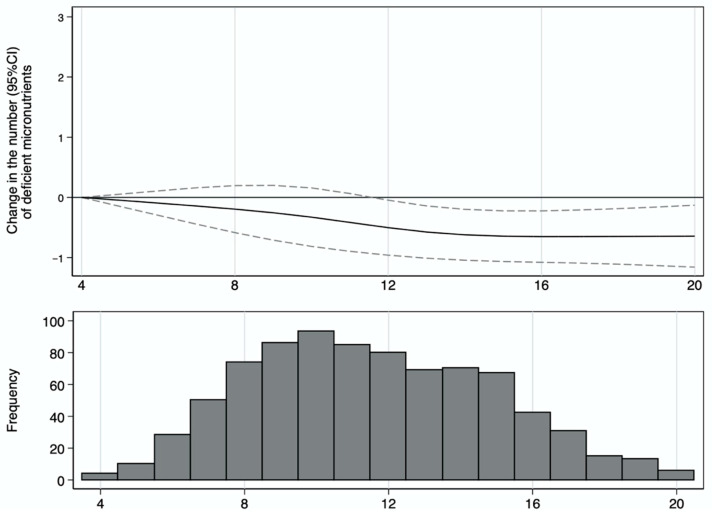
The spline above shows the change in the number of deficient micronutrients (solid line) and 95% CI (dashed line) associated with the carbohydrate quality index (CQI). The histogram below shows the frequency of participants by the CQI.

After adjusting for potential confounders, we observed that the number of inadequate intakes of micronutrients decreased as the CQI score improved, displaying an inverse linear association (*p* < 0.001), although the reduction in the number of micronutrient inadequacies for each unit of the CQI was small. This reduction became significant for values of the CQI above 11 compared to CQI values of 4 (Figure 2). The spline in Figure 2 illustrates the change in micronutrient inadequacy (solid line) and 95% CI (dashed line) associated with a one-unit increase in the CQI. The histogram in Figure 2 describes the distribution of children in our study according to their CQI. Of the study’s participants, 52% had a CQI lower or equal to 11; 31% were included in the first quartile, with the CQI ranging from 4 to 9; 21% were included in the second quartile, with the CQI ranging from 10 to 11; 27% were included in the third quartile, with the CQI ranging from 12 to 14; and 21% were included in the fourth quartile, with the CQI ranging from 15 to 20.

The OR and 95% CI for unmet EARs for ≥three micronutrients across quartiles of the CQI are shown in Table 4. We found a significant linear trend in the odds of having ≥three micronutrient inadequacies across quartiles of the CQI. Compared with children in the first quartile, those in the fourth quartile of the CQI had 0.27-fold lower odds (95% CI 0.15–0.48) of having ≥three inadequate intakes of micronutrients in the crude model. Similar numbers were observed in the adjusted models; after accounting for personal (sex, age, nutritional status, energy intake, physical activity and screen time) and family confounders (maternal age, parental knowledge about child’s nutritional recommendations, and parental attitudes towards child’s dietary habits), we found that children in the fourth quartile of the CQI had 0.22-fold lower odds (95% CI 0.10–0.48) of having ≥three micronutrient inadequacies compared with children in the first quartile.

**Figure 3:**
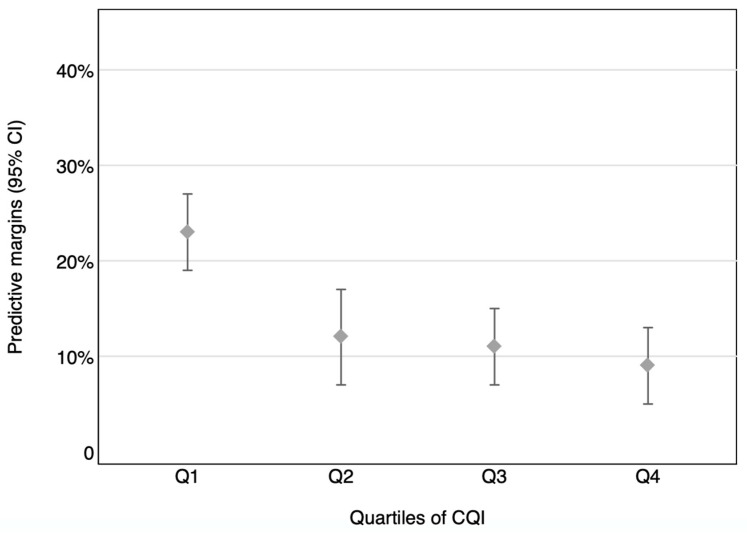
Adjusted proportions (95% CI) of children with ≥three micronutrient inadequacies by quartiles of the CQI.

We also calculated the marginal effect of the CQI on the risk of having an inadequate intake of ≥three micronutrients; in other words, the adjusted proportion of children with inadequate intakes of ≥ three micronutrients in each quartile of the CQI (Figure 3). After accounting for all potential confounders, the adjusted portions of micronutrient inadequacy were 23% (95% CI: 19–27%), 12% (95% CI; 7–17%), 11% (95% CI: 7–15%), and 9% (95% CI: 5–13%) in Q1, Q2, Q3, and Q4, respectively (*p* for trend < 0.001). Moreover, the prevalence of children with inadequate intakes of ≥three micronutrients was significantly lower in the second, third, and fourth quartiles of the CQI than in the first quartile (*p* = 0.002 for Q2 vs. Q1, *p* < 0.001 for Q3 vs. Q1, and *p* < 0.001 for Q4 vs. Q1). In additional analyses we calculated the OR and 95% CI of not meeting the EARs for ≥three micronutrients across quartiles of the different components of the CQI (Figure S1).

Finally, we performed a sensitivity analysis that took into account the supplement intake of the participants in their total micronutrient intake, and we found our results to be consistent.

## 4. Discussion

The present cross-sectional study examined the relationship between the quality of dietary carbohydrates and the risk of micronutrient intake inadequacy in Spanish children. In order to do so we used the CQI, which reflects global dietary carbohydrate quality by merging into its score the following components: the GI, total daily intake of fiber, whole grain to total grain ratio, and solid to total carbohydrates ratio. After adjusting for potential confounders, we found an inverse association between the CQI score and the risk of micronutrient intake inadequacy. To the best of our knowledge, this is the first study to analyze the association between the CQI and micronutrient inadequacy in a pediatric population.

These findings are relevant in the context of public health, as they add to previous knowledge on the relevance of carbohydrate quality in children’s diets and support the current shift in nutritional policies and guidelines.

This improvement in global nutritional adequacy, associated with an improvement in dietary carbohydrate quality, is consistent with previous studies on adults in Europe [22,31]. Moreover, these findings were also replicated in a population of Brazilian women, with presumably different dietary patterns, making the conclusions more universal [32]. In line with all of the aforementioned studies, we found a significant dose–response relationship between the CQI and nutritional adequacy, suggesting that this association is not due to residual confounding and could instead be well explained by a biological mechanism. In fact, for our results to lose their significance to an unmeasured confounder, or to a sum of them, the strength of the association between the hypothetical unmeasured confounder(s) with the outcome and the exposure ought to be 6.6 (E-value for point estimate: 6.6; confidence interval: 3.18).

In our study, the micronutrients with a greater prevalence of inadequacy were K, folate, vitamin D, vitamin E, and Ca, showing similarities with other population-based studies on children in Spain [8]; however, not all micronutrients at a higher risk of inadequate intakes in our study reflected those of other pediatric populations in Europe [49] or worldwide [6,9].

Previous evidence has reported associations between the individual components of the CQI with micronutrient adequacy. Regarding liquid carbohydrates, studies on children and adolescents have shown that sugar-sweetened beverages had, in fact, a negative effect on micronutrient intake [14,50]. Additionally, regarding the GI, a previous study showed that a higher GI was associated with a low compliance with dietary reference intakes in children and adolescents [51]. Regarding whole grain consumption, a study performed on an Italian sample of children, adolescents, and adults showed that whole grain consumption in adults was associated with significantly higher daily intakes and adequacy of several vitamins (vitamin B1, B2, and B6) as well as minerals (Fe, Ca, K, P, Zn, and Mg) compared to non-consumption. Among children, whole grain intake was associated with significantly higher intakes of Fe and Mg [52].

It is worth noting that participants across quartiles of the CQI showed similar intakes of carbohydrate, protein, and fat, supporting the idea that quality and not quantity is responsible for these variations in nutritional adequacy. In line with our expectations, participants with better scores of the CQI presented higher consumption of fruits, vegetables, legumes, nuts, and whole grains, as well as lower consumption of sugar-sweetened beverages and refined grains. These different consumption patterns are a plausible explanation for the association observed between the CQI and nutritional adequacy. As we know, whole grains, fruits, vegetables, legumes, and nuts are rich in micronutrients, and sugar-sweetened beverages provide many calories but few nutrients.

Regarding the strength of the association, the 78% relative reduction (95% CI 52–90%) in the risk of deficiencies in ≥three micronutrients observed for participants in the fourth quartile compared to those in the first quartile of the CQI represents a considerable size effect, adding epidemiological evidence in favor of a potential causal association between the CQI and nutritional adequacy.

Lastly, even though the aim of the cohort does not lie in the representativeness of its sample, the fact that our participants display relatively normal nutritional characteristics, compared to other population-based studies in Spain, supports the external validity of our results [8,53]; however, it should be noted that our participants presented rather healthy diets in general, as their intakes of fruits and vegetables, in addition to total fiber, were higher than those of the average Spanish child, as reported in other population-based studies [8]. This high quality of the diets of the SENDO participants could be attributed to different reasons, among them the fact that most of the children in our cohort come from families with highly educated parents. Moreover, participants enrolled in long-term prospective studies related to nutrition tend to be more health-conscious and motivated people [54]. In any case, the fact that the children in our study consumed healthy diets may have hampered our findings by producing smaller differences among their CQI and micronutrient inadequacies than we would have found in a population with more heterogeneous diets. The fact that the children in our study already had quite a high quality of carbohydrates in their diets does not in itself invalidate our results, quite the contrary. It is likely that our differences would have been greater had we had more participants with poorer diets.

We acknowledge that our results may not be an exact reflection of the actual magnitude of the association, which probably ranges within the confidence interval, but what should draw our attention is the potential causality between the CQI and nutritional adequacy. We acknowledge that our study has some limitations. First, given that the information collected from the participants is self-reported, including diet and anthropometric data, a misclassification bias and measurement errors cannot be entirely discarded. However, both the FFQ and the anthropometric measures have been previously validated [33,38], making measurement errors less likely. Additionally, since our data come from the FFQ, which usually slightly overestimates food intakes, the overestimation extends to nutrient intakes. This suggests that a more precise assessment of food intakes would most likely reveal a greater number of nutrient inadequacies, which are not shown here in our study. Regarding a misclassification bias, it is greatly reduced with the use of quartiles in the design of our study. Moreover, since the participants were unaware of the objective of the study, in the event of a misclassification bias the latter would be of the non-differential type, which biases the results towards the null value and makes it difficult to obtain statistically significant differences. Nevertheless, we consider the FFQ to remain the most practical and feasible tool with which to measure usual dietary intakes in epidemiological studies, since other alternative tools also come with measurement errors and present other major limitations [55].

Second, due to the observational design of our study, we cannot completely rule out the possibility of residual confounding by unknown factors. Nonetheless, the substantial amount of baseline information collected enabled us to adjust for a wide array of potential confounders. Moreover, we found an E-value of 6.6, which suggests that it is unlikely for our results to be explained simply by residual confounding. In line with this, the participants of the SENDO project are children living in a developed European country, with parents that are, for the most part, highly educated. This carries some advantages, as it better controls for potential confounding factors, such as socioeconomic variables, and most likely improves the validity of the self-reported information. Even though our sample is consequently not representative of the general Spanish population, limiting the external validity of our results, our results should be generalized on the basis of biological mechanisms and not on the representativeness of our sample [56].

Third, since our data come from the FFQ, our results show the probability of nutritional adequacy but not actual nutrient deficiency. The FFQ cannot account for variations in micronutrients in food due to variations in soil contents, nor do they capture other contributions to micronutrient status, such as sun exposure for vitamin D. These can be relevant limitations when the correlation between micronutrient inadequacy and micronutrient deficiency is poor [57,58,59].

Lastly, although the questionnaires about parental knowledge of children’s nutritional requirements and those about parental attitudes towards their child’s dietary habits have not yet been validated, previous studies have shown that they are associated with diet quality in pediatric populations [39]. Our questionnaire on physical activity has not yet been validated either.

On the other hand, our study has several strengths, including its previously validated FFQ [33] and anthropometric data [38], its extensive questionnaire, which enabled us to adjust the data for numerous potential confounders, the number of micronutrients assessed, and the importance of the issue that it tackles. Lastly, we accounted for intra-cluster correlation between siblings in all of the analyses, which is a common limitation of studies on pediatric populations.

## 5. Conclusions

In conclusion, we found that a high CQI score was associated with a lower risk of micronutrient inadequacy in children. Our results reveal that the consumption of whole grains and solid foods rich in fiber and with a low GI, at the expense of liquid sources of carbohydrates and foods rich in dietary added sugars, are important recommendations to follow in order to meet nutritional requirements in childhood. Given the pressing need to define public health policies that address the double burden of malnutrition, including carbohydrate quality in the design of nutritional strategies represents a promising line of action. More research is needed to confirm this association in other pediatric populations with different diets.

## Figures and Tables

**Table 1:** Characteristics of participants and their family in the SENDO project by quartiles of the CQI. Numbers are mean (SD) or N (%).

	CQI				
	Q1	Q2	Q3	Q4	*p* for Trend
n	252	178	219	174	
Range of CQI	4 to 9	10 to 11	12 to 14	15 to 20	
Mother’s Characteristics					
Maternal age (years)	39.58 (4.81)	39.93 (4.10)	40.32 (4.19)	39.86 (4.13)	0.32
Maternal age (years), %					0.65
<35	33 (13.10)	16 (8.99)	23 (10.50)	24 (13.79)	
35–40	107 (42.46)	74 (41.57)	80 (36.53)	64 (36.78)	
40–45	81 (32.14)	67 (37.64)	92 (42.01)	69 (39.66)	
>45	31 (12.30)	21 (11.80)	24 (10.96)	17 (9.77)	
Maternal high education, %	199 (78.97)	144 (80.90)	181 (82.65)	145 (83.33)	0.20
Number of Children, %					<0.001
1	32 (12.70)	17 (9.55)	25 (11.42)	33 (18.97)	
2	123 (48.81)	85 (47.75)	125 (57.08)	101 (58.05)	
3–4	79 (31.35)	63 (35.39)	62 (28.31)	36 (20.69)	
5 or more	18 (7.14)	13 (7.30)	7 (3.20)	4 (2.30)	
Family history of obesity, %	51 (20.40)	37 (21.02)	45 (20.64)	24 (14.04)	0.16
Parental Attitudes Towards Child’s Dietary Habits, %					<0.001
Low score (<40%)	29 (11.51)	7 (3.93)	3 (1.37)	4 (2.30)	
Medium score (40–70%)	116 (46.03)	60 (33.71)	62 (28.31)	30 (17.24)	
High score (>70%)	107 (42.46)	111 (62.36)	154 (70.32)	140 (80.46)	
Parental Knowledge About the Child’s Nutritional Recommendations, %					<0.001
Low score (<40%)	71 (28.17)	50 (28.09)	41 (18.72)	24 (13.79)	
Medium score (40–70%)	157 (62.30)	110 (61.80)	144 (65.75)	115 (66.09)	
High score (>70%)	24 (9.52)	18 (10.11)	34 (15.53)	35 (20.11)	
Children’s Characteristics					
Sex (female), %	124 (49.21)	93 (52.25)	103 (47.03)	84 (48.28)	0.64
Age (years)	5.08 (0.86)	5.13 (0.90)	4.93 (0.82)	4.83 (0.76)	<0.001
Race (white), %	242 (96.03)	171 (96.07)	216 (99.08)	165 (94.83)	0.99
Gestational Age (Weeks), %					0.04
<38	36 (14.34)	25 (14.12)	35 (16.06)	17 (9.88)	
38 to 40	109 (43.43)	77 (43.50)	81 (37.16)	64 (37.21)	
>40	106 (42.23)	75 (42.37)	102 (46.79)	91 (52.91)	
Birthweight (g)	3216 (568.72)	3211 (570.72)	3273 (477.50)	3250 (475.23)	0.24
Birthweight (g), %					0.23
<2500	27 (10.76)	20 (11.30)	17 (7.80)	12 (6.98)	
2500–3000	59 (23.51)	35 (19.77)	43 (19.72)	39 (22.67)	
3000–3500	92 (36.65)	69 (38.98)	94 (43.12)	70 (40.70)	
3500–4000	60 (23.90)	48 (27.12)	48 (22.02)	43 (25.00)	
>4000	13 (5.18)	5 (2.82)	16 (7.34)	8 (4.65)	
Breastfeeding Duration (Months), %					<0.001
No breastfeeding	49 (19.44)	37 (20.79)	34 (15.53)	17 (9.77)	
<6	90 (35.71)	50 (28.09)	57 (26.03)	40 (22.99)	
6 to 12	64 (25.40)	48 (26.97)	58 (26.48)	42 (24.14)	
>12	49 (19.44)	43 (24.16)	70 (31.96)	75 (43.10)	
Child’s Position Among Siblings, %					0.09
The oldest/singletons	70 (27.78)	69 (38.76)	87 (39.73)	72 (41.38)	
2nd/3, 2nd or 3rd/4	48 (19.05)	36 (20.22)	31 (14.16)	11 (6.32)	
The youngest or beyond the fourth	134 (53.17)	73 (41.01)	101 (46.12)	91 (52.30)	
Z-score of the BMI	0.18 (1.12)	(−) 0.08 (1.14)	0.12 (1.20)	(−) 0.05 (1.10)	0.16
Nutritional Status, %					0.08
Low weight	32 (12.70)	32 (17.98)	34 (15.53)	27 (15.52)	
Normal weight	184 (73.02)	123 (69.10)	154 (70.32)	136 (78.16)	
Overweight/obese	36 (14.29)	23 (12.92)	31 (14.16)	11 (6.32)	
Moderate–vigorous physical activity (h/day)	1.00 (0.70)	1.02 (0.72)	1.21 (0.84)	1.26 (0.80)	<0.001
Screen time (h/day)	1.19 (0.90)	1.07 (0.89)	1.14 (1.21)	0.97 (0.70)	0.05

Simple linear regression models for continuous variables using the median CQI in each quartile as a continous variable. Linear trend chi-squared tests for categorical variables.

**Table 2:** Nutritional characteristics of participants according to quartiles of the CQI. Mean values and standard deviations; number of participants and percentages.

	CQI				
	Q1	Q2	Q3	Q4	*p* for Trend
n	252	178	219	174	
TEI (Kcal/d)	1923 (445.5)	2143 (469.9)	2072 (501.0)	2042 (436.2)	0.01
Carbohydrate intake (% of TEI)	42.84 (4.94)	42.47 (5.11)	43.76 (5.31)	44.60 (5.38)	<0.001
Protein intake (% of TEI)	17.09 (1.96)	17.09 (2.40)	17.20 (2.18)	16.84 (2.13)	0.40
Fat intake (% of TEI)	40.06 (5.00)	40.44 (5.21)	39.04 (5.40)	38.56 (5.33)	<0.001
SFA intake (% of TEI)	11.73 (2.13)	11.67 (2.07)	10.87 (2.04)	10.28 (2.02)	<0.001
PUFA intake (% of TEI)	4.72 (1.34)	4.70 (0.94)	4.68 (1.03)	4.64 (1.03)	0.40
MUFA intake (% of TEI)	15.25 (3.32)	15.53 (3.60)	15.08 (3.56)	15.46 (3.50)	0.90
Fibre intake (g/d)	15.91 (4.19)	20.21 (3.87)	22.51 (5.42)	27.17 (6.10)	<0.001
KIDMED score (p50 (IQR))	5 (4–6)	6 (5–7)	7 (6–8)	7 (6–8)	< 0.001
Food Groups					
Vegetables (g/d)	132.1 (79.1)	192.7 (95.3)	209.7 (103.8)	254.2 (116.3)	<0.001
Fruits (g/d)	266.3 (157.6)	333.1 (155.0)	415.9 (216.3)	516.2 (247.2)	<0.001
Legumes (g/d)	24.42 (12.55)	31.51 (13.99)	22.59 (16.61)	42.04 (24.93)	<0.001
Dairy (g/d)	528.5 (235.3)	518.3 (260.6)	471.4 (225.2)	397.3 (225.0)	<0.001
Cereals (g/d)	77.28 (37.02)	76.73 (40.67)	78.41 (41.59)	74.32 (34.31)	0.59
Potatoes (g/d)	14.69 (15.13)	18.82 (16.93)	20.38 (24.17)	21.90 (17.78)	<0.001
Meat (g/d)	133.43 (42.44)	146.15 (44.36)	133.2 (47.17)	119.2 (46.20)	<0.001
Fish (g/d)	30.77 (15.06)	36.18 (17.77)	36.75 (16.83)	38.45 (16.38)	<0.001
Nuts (g/d)	3.08 (4.40)	4.46 (6.00)	5.80 (6.53)	8.98 (11.88)	<0.001
Bakery and sweets (g/d)	77.32 (44.67)	95.75 (71.37)	79.44 (49.02)	79.44 (49.02)	0.16
Sugar-sweetened beverages (g/d)	57.67 (95.31)	52.24 (66.83)	37.23 (70.77)	23.93 (39.29)	<0.001
Fast Food (g/d)	58.72 (25.69)	64.72 (28.46)	59.84 (31.67)	52.33 (25.32)	0.01
Eggs (g/d)	18.24 (11.17)	19.83 (7.18)	20.96 (10.90)	20.26 (7.86)	0.01
Olive oil (g/d)	9.96 (11.99)	13.08 (15.92)	9.95 (12.40)	8.21 (11.95)	0.05
Other fats (g/d)	3.22 (4.55)	2.52 (3.67)	2.48 (3.13)	2.22 (3.43)	0.009

TEI: total energy intake; simple linear regression models for continuous variables using the median CQI in each quartile as a continous variable.

**Table 3:** Energy-adjusted intake of micronutrients by quartiles of the CQI. Mean (SD).

	CQI				
	Q1	Q2	Q3	Q4	*p* for Trend
n	252	178	219	174	
Micronutrients					
Vitamin A (equiv Retinol) (µg/d)	922 (26.7)	1008 (31.7)	1143 (28.4)	1255 (31.8)	0.001
Vitamin C (mg/d)	106 (3.37)	124 (4.00)	155 (3.59)	184 (4.03)	0.001
Vitamin D (µg/d)	2.98 (0.11)	2.93 (0.13)	3.27 (0.12)	3.41 (0.13)	0.002
Vitamin E (mg/d)	8.02 (0.17)	7.83 (0.20)	8.36 (0.18)	9.36 (0.20)	0.001
Vitamin B1 (mg/d)	1.40 (0.01)	1.40 (0.02)	1.47 (0.02)	1.50 (0.02)	0.001
Vitamin B2 (mg/d)	2.03 (0.03)	2.05 (0.04)	2.08 (0.03)	2.02 (0.04)	0.93
Vitamin B3 (mg/d)	33.6 (0.47)	35.6 (0.55)	36.6 (0.50)	38.1 (0.55)	0.001
Vitamin B6 (mg/d)	2.08 (0.26)	2.21 (0.03)	2.37 (0.03)	2.58 (0.03)	0.001
Folic acid (µg/d)	262 (4.42)	282 (5.25)	318 (4.70)	355 (5.27)	0.001
Vitamin B12 (µg/d)	4.63 (0.08)	4.63 (0.10)	4.84 (0.88)	4.60 (0.10)	0.68
Ca (mg/d)	1205 (15.8)	1155 (18.7)	1165 (16.8)	1137 (18.8)	0.01
I (µg/d)	111 (1.45)	109 (1.71)	110 (1.54)	107 (1.72)	0.18
Fe (mg/d)	12.9 (0.12)	13.6 (0.14)	14.4 (0.13)	15.3 (0.14)	0.001
P (mg/d)	1667 (40.6)	1655 (48.2)	1788 (43.2)	1841 (48.5)	0.001
Mg (mg/d)	269 (2.37)	289 (2.81)	310 (2.52)	341 (2.82)	<0.001
Se (µg/d)	71.0 (0.83)	70.4 (0.99)	73.6 (0.89)	72.8 (0.99)	0.03
Zn (mg/d)	9.02 (0.12)	9.62 (0.15)	9.95 (0.13)	10.07 (0.15)	<0.001
Cr (µg/d)	62.3 (1.25)	66.1 (1.49)	67.0 (1.33)	74.7 (1.50)	<0.001
K (mg/d)	3073 (34.4)	3278 (40.8)	3542 (36.6)	3848 (41.0)	<0.001
Na (mg/d)	3060 (53.2)	2994 (56.7)	2886 (63.5)	2886 (63.5)	0.01

Simple linear regression models for continuous variables using the median CQI in each quartile as a continous variable.

**Table 4:** Odds Ratio (OR) and 95% confidence interval (CI) for inadequate intake of ≥3 micronutrients associated with quartiles of CQI.

	CQI				*p* for Trend
	Q1	Q2	Q3	Q4	
n	252	178	219	174	
Crude	1.00 (ref)	0.29 (0.16–0.51)	0.29 (0.17–0.51)	0.27 (0.15–0.48)	<0.001
Multivariate adjusted model 1	1.00 (ref)	0.34 (0.17–0.67)	0.30 (0.16–0.54)	0.24 (0.12–0.48)	<0.001
Multivariate adjusted model 2	1.00 (ref)	0.33 (0.16–0.67)	0.29 (0.15–0.54)	0.22 (0.11–0.47)	<0.001
Multivariate adjusted model 3	1.00 (ref)	0.33 (0.16–0.66)	0.28 (0.15–0.54)	0.22 (0.10–0.48)	<0.001

Model 1: adjusted for sex (male vs. female), age (continuous), nutritional status (low weight, normal weight, and overweight/obesity), and energy intake (continuous); Model 2: additionally adjusted for maternal age (continuous), number of children (1, 2, 3–4, 5 or more), breastfeeding duration (none, <6 months, 6–12 months, and >12 months), birth weight (continuous), parental knowledge about nutritional recommendations (low, medium, and high score) for children, and parental attitudes towards child’s dietary habits (low, medium, and high score); and Model 3: additionally adjusted for moderate–vigorous physical activity (continuous) and screen time (continuous).

## Data Availability

Data are available on request.

## Supplementary Materials

The following supporting information can be downloaded at: https://www.mdpi.com/article/10.3390/children10101711/s1, Figure S1: Odds ratio and 95% CI of inadequate intake of ≥three micronutrients associated with quartiles of each component of the CQI; Table S1: Criteria used to calculate the carbohydrate quality index (CQI); Table S2: Parameters of carbohydrate quality intake across quartiles of the CQI. Mean (SD); Table S3: Prevalence of inadequate micronutrient intake according to quartiles of the CQI (percentages).
