# Supplementary material for: Association between the Carbohydrate Quality Index (CQI) and Nutritional Adequacy in a Pediatric Cohort: The SENDO Project
Source: Children (Basel). 2023 Oct 20;10(10):1711. doi: 10.3390/children10101711 (PMC10605036; doi:10.3390/children10101711)
Supplement: Supplementary file 1 [file children-10-01711-s001.zip › children-2664282-supplementary.pdf]

SUPPLEMENTARY MATERIAL

Table S1. Criteria used to calculate the carbohydrate quality index (CQI).

Components of the carbohydrate quality index (CQI)	Index range (points)	Criteria for minimum index	Criteria for maximum index
**Dietary fiber intake (g/d)**	1-5	Minimum dietary fiber (first quintile)	Maximum dietary fiber intake (fifth quintile)
**Glycemic index**	1-5	Maximum glycemic index (first quintile)	Minimum glycemic index (fifth quintile)
**Ratio of whole grains: (whole grains + refined grains of their products)**	1-5	Minimum value of this ratio (first quintile)	Maximum value of this ratio (fifth quintile)
**Ratio of solid carbohydrates (g): (solid carbohydrates (g) + liquid carbohydrates (g))**	1-5	Minimum value of this ratio (first quintile)	Maximum value of this ratio (fifth quintile)
**Total index (range)**	**4-20**		

Table S2. Parameters of carbohydrate quality intake across quartiles of CQI. Mean (sd)

	CQI			
	Q1	Q2	Q3	Q4
Fibre intake (g/d)	15.91 (4.19)	20.21 (3.87)	22.51 (5.42)	27.17 (6.10)
Glycemic index	50.5 (3.6)	49.4 (4.0)	48.8 (3.3)	47.6 (3.5)
Carbohydrate intake from whole grains (g/d)	0.62 (2.52)	1.45 (3.29)	7.02 (10.18)	11.99 (10.30)
Carbohydrate intake from refined grains (g/d)	74.56 (28.73)	74.67 (31.37)	67.61 (29.05)	56.88 (23.88)
Solid carbohydrate intake (g/d)	171.7 (46.5)	194.6 (45.7)	198.5 (52.5)	206.6 (45.9)
Liquid carbohydrate intake (g/d)	34.4 (17.2)	31.8 (17.7)	27.9 (17.0)	19.9 (13.3)

Table S3. Prevalence of inadequate micronutrient intake according to quartiles of CQI (percentages).

	CQI			
	**Q1**	**Q2**	**Q3**	**Q4**
**n**	**252**	**178**	**219**	**174**
**% Inadequate Intake**				
**Vitamin A (equiv Retinol)**	2.4	0.0	0.0	0.0
**Vitamin C**	1.2	0.0	0.0	0.0
**Vitamin D**	100	100	100	100
**Vitamin E**	35.7	24.7	21.5	14.9
**Vitamin B1**	0.0	0.0	0.0	0.0
**Vitamin B2**	0.0	0.0	0.0	0.0
**Vitamin B3**	0.0	0.0	0.0	0.0
**Vitamin B6**	0.0	0.0	0.0	0.0
**Folic Acid**	11.1	2.3	0.9	0.6
**Vitamin B12**	0.0	0.0	0.0	0.6
**Ca**	17.9	11.8	11.9	13.8
**I**	9.1	3.9	3.2	5.7
**Fe**	0.0	0.0	0.0	0.0
**P**	0.0	0.0	0.0	0.0
**Mg**	0.0	0.0	0.0	0.0
**Se**	0.0	0.0	0.0	0.0
**Zn**	0.0	0.0	0.0	0.0
**Cr**	0.8	0.0	0.0	0.0
**K**	26.2	5.6	5.9	2.3
**Na**	0.4	0.0	1.37	0.6

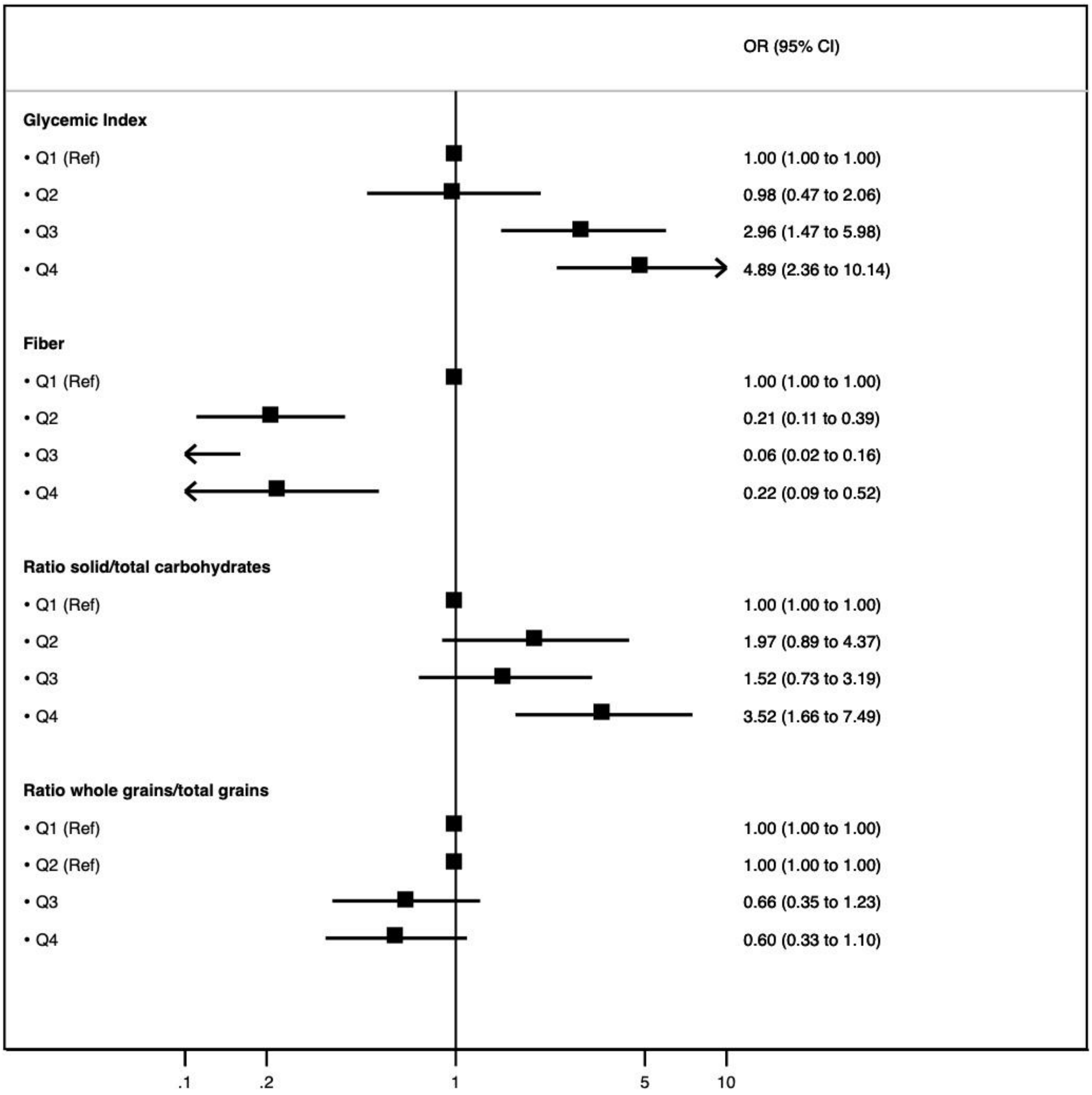

Figure S1. Odds Ratio and 95% CI of inadequate intake of ≥3 micronutrients associated with quartiles of each component of the CQI.
